# Recurrent ulcerative tubular apocrine adenoma of the eyelid mimicking eyelid malignancy: A case report and literature review

**DOI:** 10.1016/j.ijscr.2025.110929

**Published:** 2025-01-23

**Authors:** Ashwaq Y. Asiri, Hamad Alsulaiman, Hind M. Alkatan, Rawan N. Althaqib

**Affiliations:** a Department of Ophthalmology, College of Medicine, King Khalid University, Abha, Saudi Arabia; b King Khaled Eye Specialist Hospital, Riyadh, Saudi Arabia; c Departments of Ophthalmology and Pathology, College of Medicine, King Saud University, Riyadh, Saudi Arabia; d King Saud University Medical City, King Saud University, Riyadh, Saudi Arabia

**Keywords:** Apocrine, Tubular adenoma, Recurrent, Eyelid, Case report

## Abstract

**Introduction:**

The eyelid is a common site for cutaneous tumors; however, tubular apocrine adenoma is a rare presentation. The clinical presentation is variable and surgical excision is effective with low risk for recurrence.

**Presentation of case:**

We report a 40-year-old female with recurrent tubular apocrine adenoma of eyelid mimicking eyelid malignancy. Diagnostic incisional biopsy demonstrated dermal benign tubular-like proliferations lined by bilayer of apocrine epithelium within a background of hyalinized stroma.

**Discussion:**

Isolated tubular apocrine adenoma of the eyelid is rare with individual cases being found in the literature. Previously reported recurring apocrine eyelid tumors were involving older patients than our case and the recurrence occurred within 2–3 years.

**Conclusion:**

Our case is unique as the patient was younger and the recurrence occurred after a longer period. Our report provides insight into the clinical features, histopathologic characteristics and treatment of tubular apocrine adenoma of the eyelid.

## Highlights

• Tubular apocrine adenoma (TAA) is a benign skin adnexal neoplasm with apocrine differentiation.
• Histopathologically, it shows proliferation of tubules with double layer of apocrine epithelium.
• TAA of the eyelid is exceedingly rare, and recurrence was reported in only 2 patients in their sixties.
• Our TAA case is unique because of her age, late recurrence, and suspicious presentation for malignancy.
• TAA (even though is rare) should be included in the differential diagnosis of eyelid lesions.

## 1 Introduction

Eyelid neoplasms comprise a variety of benign and malignant growths. Majority of these growths are benign in nature and constitute 82–98 % of all neoplasms [1]. There is a wide racial and geographical variations related to the incidence of eyelid tumors [2]. One of the benign adnexal neoplasms of the eyelid with apocrine differentiation is Tubular Apocrine Adenoma (TAA), which is a rare tumor of the eyelid. It is considered within the spectrum of tubular adenomas. TAAs are usually found in the scalp area, the face, axilla, and anogenital area [3]. Histopathological features of this unique tumor include a well-defined proliferation of tubules surrounded by a bilayer of apocrine epithelium against a background of hyalinized stroma. It's interesting to note that it can also show up as a component of other apocrine gland tumors, like apocrine cystadenoma and syringocystadenoma papilliferum [4]. Isolated occurrences of tubular apocrine adenoma in the eyelid are exceedingly rare, with only few documented cases in existing literature [[3], [4], [5], [6], [7], [8], [9]]. Furthermore, it is unusual for apocrine tumors to present as recurring ulcerative lesions [8]. In this comprehensive report, we present a unique case of recurrent ulcerative tubular apocrine adenoma arising from the apocrine gland mimicking eyelid malignancy. A literature review was performed on PubMed and Google Scholar for the period 1963 to 2023 and search results were limited to articles published in the English-written language. Our keywords were used in the search. This case report has been also prepared and reported in line with the SCARE criteria [10].

## 2 Presentation of case

A 40-year-old-female was referred to our tertiary eye hospital for evaluation of a recurrent left upper eyelid painless lesion with a recent concern significant for generalized itchiness and recurrent bleeding throughout the previous 3 years in total. Of note her medical history was extensive and included poorly controlled insulin dependent type 2 diabetes, myasthenia gravis, hypothyroidism, and abnormal liver function tests in the form of elevated liver enzymes. In addition to this, she experienced a deep vein thromboembolism (DVT) event, which is currently managed by warfarin anticoagulation.

The history of this lesion goes back to 13 years ago with history of previous surgical excision of an identical mass in the same location 10 years earlier elsewhere. Unfortunately, no available histopathological information nor diagnosis about the nature of the formerly excised mass.

**Fig. 1:**
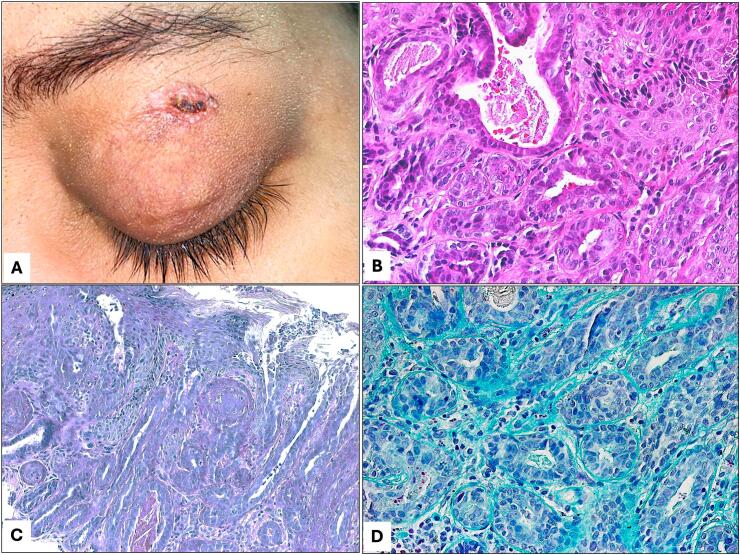
A: The clinical appearance of the recurrent left upper lid lesion. B: The histopathological appearance of the tubular proliferation of bilayer apocrine epithelium (Original magnification ×400 Hematoxylin and eosin). C: The lesion with overlying skin changes and collection of PAS positive material (Original magnification ×200 Periodic acid Schiff). D: The same glandular tubules with Alcian blue staining (Original magnification ×400 Alcian blue).

Upon examination, the patient had a left upper lid skin lesion measuring 9 × 6 mm oval, well-demarcated, red-colored, firm, non-tender, ulcerated nodule with overlying crust formation. The mass was not involving the lid margin and there were no palpable pre-auricular lymph nodes (Fig. 1A). The remainder of the eye examination was unremarkable apart from diabetic retinopathy and cataract changes in both eyes.

Incisional biopsy was performed for diagnostic purpose taking into consideration the recurring nature and the presence of ulceration. The histopathological examination showed benign dermal tubular-like proliferations lined by bi-layered epithelium of apocrine nature within a background of hyalinized stroma. The overlying skin showed focal ulceration and adjacent hyperkeratosis. The final diagnosis was TAA (Fig. 1B–D). Further management plan for full thickness excision of the lesion with safety margin was discussed with the patient, who expressed a lack of interest in immediate surgical intervention. The patient was given a follow up appointment for planned surgical excision, which was discussed extensively with her but was lost to follow up for over 2 years. She finally underwent a full-thickness excision with a 2 mm safety margin as the preferred treatment 34 months after the diagnosis of her recurrent lesion. No further recurrence has been noted since then with a limited follow-up period of 2 months.

## 3 Discussion

Isolated TAA of the eyelid is an extremely uncommon benign tumor of apocrine origin with only few reported occurrences in the literature to date. Out of the reported cases after extensive PubMed literature search (Table 1), six cases—two females and 4 male patients—with an average age of 45 years (range from 23 to 63) showed clinical signs of a well-circumscribed nodule affecting either the upper or lower eyelid. Notably, we found only two reported cases that have shown recurrence [3,8]. Our patient exhibited a well-defined nodule on the upper eyelid, which was ulcerated and recurred after a long gap of 10 years. In the previous case reports of recurring apocrine eyelid tumor, both patients were of older age (63 years) and the recurrence occurred after 2–3 years [3,8]. Our patient comparatively was younger with much longer-term recurrence. In cases where recurrence occurred after primary lesion excision, including our own, patients underwent full-thickness excision of the lesion with a safety margin as the treatment. No recurrence was observed following this approach [3,8].

**Table 1:** Summary of the 6 reported Tubular apocrine adenoma cases involving the eyelids including our current case.

Case	Age (years)	Gender	Location	Duration	Treatment	Recurrence	Treatment of recurrence
Stokes et al. (2005) [3]	63	Female	Lower lid	2 years (Primary lesion)	Excisional biopsy	Yes (6 months duration)	Excised with a 2 mm margin of normal skin
Barker-Griffith et al. (2006) [6]	40	Male	Left lower eyelid	1 year	Excisional biopsy	None	Not applicable
Raven et al. (2016) [7]	42	Male	Left Medial commissure	8 years	Excisional biopsy	None	Not applicable
Fu et al. (2018) [9]	23	Male	Left upper lid	7 months	Excisional biopsy	None	Not applicable
Abbeel et al. (2018) [5]	38	Female	Left upper eyelid	1 year	En bloc excision	None	Not applicable
Eiger-Moscovich et al. (2019) [8]	63	Male	Left upper eyelid	3 years (Primary lesion)	Excisional biopsy	Yes	full-thickness Excision with frozen section control of margins followed by cryotherapy to the surgical margins
Asiri et al. Current case	40	Female	Left upper eyelid	3 years	Excisional biopsy	Yes (after 10 years duration)	Full-thickness excision with 2 mm margin of normal skin

Although TAA is uncommon when it is isolated and pure, it might resemble papillary eccrine adenoma morphologically, thus creating clinical and histopathological diagnostic challenges. To help differentiating between TAA and the later entity, papillary eccrine adenoma is more frequently seen in the extremities than TAA, which mainly affects the skin of the head and neck region [11]. Papillary eccrine adenoma is also characterized by intraluminal papillary or micropapillary growth patterns and eccrine differentiation without apocrine decapitation secretion [12]. Tumors that exhibit traits of both papillary eccrine adenoma and TAA, however, have been recognized as having hybrid features [13].

Regarding the treatment of apocrine adenomas of the eyelid, complete surgical excision of the lesion with safety margin, followed by reconstruction of the resultant defect (accordingly if required) remains the method of choice, which was carried on for our patient [5,14].

## 4 Conclusion

TAA of the eyelid is a rare pathology that should be considered in the differential diagnosis by ophthalmologists who deal with eyelid skin lesions. Our case highlights the clinical features, recurrence behavior, and histopathological characteristics of recurrent TAA of the eyelid. It also elicits the frequent morphological overlap encountered in various sweat gland tumors. These cases may have suspicious signs that might be indicative for malignancy. Thus, obtaining biopsies for histopathological examination is crucial for diagnosing such rare entities and delivering the appropriate management.

## Consent

Written informed consent was obtained from the patient for publication and any accompanying images. A copy of the written consent is available for review by the Editor-in-Chief of this journal on request.

## Ethical approval

An ethical approval is not required for case reports as per the regulations of the Institutional Review Board. However, information was obtained and reported in a manner that was compliant with the standards set forth by the Health Insurance Portability and Accountability Act, and the Declaration of Helsinki as amended in 2013.

## Provenance and peer review

Not commissioned, externally peer reviewed.

## Source of funding

This research did not receive any specific grant from funding agencies in the public, commercial, or not-for-profit sectors.

## Authors' contributions

**Ashwaq Y Asiri:** acquisition of data and drafting of the manuscript; **Hamad Alsulaiman**: concept and design of the study; **Hind M. Alkatan:** Histopathological diagnosis; critical review of the manuscript for submission; **Rawan N Althaqib:** the final approval of the version.

## Research registration

Not applicable.

## Declaration of competing interest

None.
